# Identification and expression profiling analysis of calmodulin-binding transcription activator genes in maize (*Zea mays* L.) under abiotic and biotic stresses

**DOI:** 10.3389/fpls.2015.00576

**Published:** 2015-07-28

**Authors:** Runqing Yue, Caixia Lu, Tao Sun, Tingting Peng, Xiaohua Han, Jianshuang Qi, Shufeng Yan, Shuanggui Tie

**Affiliations:** ^1^Henan Academy of Agricultural SciencesZhengzhou, China; ^2^The Henan Provincial Key Lab. of Maize BiologyZhengzhou, China; ^3^Zhejiang Provincial Key Laboratory for Genetic Improvement and Quality Control of Medicinal Plants, College of Life and Environmental Sciences, Hangzhou Normal UniversityHangzhou, China

**Keywords:** abiotic stress, calmodulin, biotic stress, *CAMTA* gene family, maize

## Abstract

The calmodulin-binding transcription activators (CAMTA) play critical roles in plant growth and responses to environmental stimuli. However, how *CAMTAs* function in responses to abiotic and biotic stresses in maize (*Zea mays* L.) is largely unknown. In this study, we first identified all the *CAMTA* homologous genes in the whole genome of maize. The results showed that nine *ZmCAMTA* genes showed highly diversified gene structures and tissue-specific expression patterns. Many *ZmCAMTA* genes displayed high expression levels in the roots. We then surveyed the distribution of stress-related *cis*-regulatory elements in the −1.5 kb promoter regions of *ZmCAMTA* genes. Notably, a large number of stress-related elements present in the promoter regions of some *ZmCAMTA* genes, indicating a genetic basis of stress expression regulation of these genes. Quantitative real-time PCR was used to test the expression of *ZmCAMTA* genes under several abiotic stresses (drought, salt, and cold), various stress-related hormones [abscisic acid, auxin, salicylic acid (SA), and jasmonic acid] and biotic stress [rice black-streaked dwarf virus (RBSDV) infection]. Furthermore, the expression pattern of *ZmCAMTA* genes under RBSDV infection was analyzed to investigate their potential roles in responses of different maize cultivated varieties to RBSDV. The expression of most *ZmCAMTA* genes responded to both abiotic and biotic stresses. The data will help us to understand the roles of CAMTA-mediated Ca^2+^ signaling in maize tolerance to environmental stresses.

## Introduction

The divalent ion of calcium (Ca^2+^) is probably the most ubiquitous second messenger in eukaryotes (Galon et al., 2010b). Ca^2+^ plays a key role in regulating many aspects of the organism's life cycle and functions as a signal messenger to respond to environmental stimuli (Ikura et al., 2002; Kudla et al., 2010). Ca^2+^ signals are sensed and integrated into appropriate physiological responses depending on their different loading statuses (Du et al., 2009; Dodd et al., 2010; Reddy et al., 2011). In plants, three major types of Ca^2+^-sensor proteins, including calmodulin (CaM)/CaM-like proteins, calcium-dependent protein kinases, and calcineurin B-like proteins, are involved in the mechanism of Ca^2+^-dependent transcription regulation (Reddy, 2001), and Ca^2+^ can also bind to and control certain transcription factors (TFs) (Poovaiah et al., 2013). The novel CaM/CaM-like family, which belongs to the superfamily of EF-hand Ca^2+^-binding proteins, are important Ca^2+^ signal transducers in eukaryotic cells of various plant species (Finkler et al., 2007). CaM/CaM-like proteins in plants regulate a large number of downstream targets including TFs, protein kinases, phosphatases, metabolic enzymes, ion channels, ion exchangers, ion pumps, and cytoskeletal proteins (Yang and Poovaiah, 2003; Bouche et al., 2005; Poovaiah et al., 2013).

To elucidate the mechanisms underlying Ca^2+^/CaM-mediated gene expression regulation, a family of CaM-binding transcription activators (CAMTAs, also known as signal responsive proteins) has been reported in multi-cellular eukaryotes from plants to humans (Bouche et al., 2002; Yang and Poovaiah, 2002; Finkler et al., 2007). Six members with highly conserved functional domains are identified as CAMTAs in the model plant *Arabidopsis* (Bouche et al., 2002; Finkler et al., 2007). A typical CAMTA protein contains a CG-1 homology DNA-binding domain at the N-terminus, a TIG domain (implicated in nonspecific DNA contacts in TFs), three ankyrin repeats, which are present as tandem repeat modules of about 33 amino acids, and five putative variable CaM-binding motifs named as the IQ motif (Reddy et al., 2000; Bouche et al., 2002; Song et al., 2006). Recent investigations of fly and mammals in addition to plants indicate diverse functions of these domains in gene expression regulation (Han et al., 2006; Long et al., 2014). The CAMTAs in fly may mediate a long-term feedback regulation of the activity of Ca^2+^ stimulating targets to prevent extra Ca^2+^ influx (Han et al., 2006). In humans, HsCAMTA2 is reported to bind to a homeodomain-type TF as a co-activator (Song et al., 2006).

Transcriptional regulation of physiological and morphological changes by controlling the endogenous level of receptor proteins is a critical strategy of plants to survive in and adapt to challenging environments (Wang et al., 2015). CAMTAs participate in gene expression regulation by binding to the *cis*-elements in the promoter regions of numerous target genes. The CAMTA-binding *cis*-element (G/A/C)CGCG(C/G/T) was first identified in *Arabidopsis* by Yang and Poovaiah (2002). Then, another *cis*-element, (A/C)CGTGT, which encompasses a classic abscisic acid (ABA)-responsive element (ABRE: ACGTGT), was confirmed by the CAMTA homologs in rice (*Oryza sativa*) (Choi et al., 2005; Kaplan et al., 2006).

The expression of *CAMTA* genes responds to both hormonal stimuli, such as auxin, ethylene, ABA and salicylic acid (SA), and environmental stresses (Yang and Poovaiah, 2002; Galon et al., 2008; Yang et al., 2012). In *Arabidopsis*, calmodulin-binding transcription activator 1 (*AtCAMTA1*) plays a role with auxin signaling responses, and its expression pattern is changed significantly by exogenous auxin with a cell-specific manner (Galon et al., 2010a). AtCAMTA1 is also involved in expression regulation of a broad spectrum of membrane integrity response genes by generating ABA response to drought stress (Pandey et al., 2013). The *camta1* mutant displays a high-susceptibility to induced osmotic stress, and several stress-responsive gene promoters are enriched with a CAMTA recognition *cis*-element (Pandey et al., 2013). AtSR1, a Ca^2+^/calmodulin-binding transcription factor (AtCAMTA3), functions as a regulator of SA-mediated immune response by interacting with the promoter of *EDS1* gene and repressing its expression (Du et al., 2009). Furthermore, elevated SA contents in *atsr1* mutant inhibit biosynthesis of jasmonate (JA), and the SA-JA crosstalk is critical for AtSR1-mediated herbivore-induced wound responses (Qiu et al., 2012). In tomato (*Solanum lycopersicum*), the expression of *SlSR4*/*CAMTA4* is up-regulated by SA (Yang et al., 2013).

Recently, some AtCAMTAs were reported to be involved in abiotic stress responses, and the *camta1/camta3* double mutant has impaired freezing tolerance, suggesting a possible connection of calcium/calmodulin signaling with cold-regulated gene expression (Doherty et al., 2009). The first evidence for the involvement of Ca^2+^/CaM-mediated signaling in ethylene action was reported in tobacco. NtER1, an early ethylene up-regulated protein, binds to CaM with high affinity in a Ca^2+^-dependent manner and acts as a trigger for senescence and death (Yang and Poovaiah, 2000). AtSR1 regulates ethylene-induced senescence by directly binding to the *ETHYLENE INSENSITIVE3* (*EIN3*) promoter region *in vivo* (Nie et al., 2012). Seven *SR/CAMTAs* have been cloned in tomato, and the *SlSR* gene expressions are influenced by ethylene signaling during different fruit ripening and storage stages (Yang et al., 2012, 2013). The expression of *SlSR1L* in the detached leaves and whole plants is significantly up-regulated by drought stress, and silencing of *SlSR1L* leads to decreased drought stress tolerance (Li et al., 2014).

Maize (*Zea mays* L.) is one of the most important crops providing food and biofuel to large populations. Growth and productivity of maize plants are suffer from various abiotic and biotic stresses, and the adaptive mechanisms are an essential base to survive in these challenging environmental conditions (Verslues et al., 2006). After first being identified in tobacco, the well-characterized CAMTA family has been reported in various plant species, including *Arabidopsis*, rice, tomato, grape (*Vitis vinifera*), and soybean (*Glycine max*) (Yang and Poovaiah, 2000; Koo et al., 2009; Yang et al., 2013; Shangguan et al., 2014; Wang et al., 2015). However, information and expression patterns of responses of *CAMTA* family genes to abiotic and biotic stresses in maize are largely unknown. To date, most work on *CAMTA* genes has focused on *Arabidopsis* and tomato. Identification and expression analysis of *CAMTA* genes in maize may provide preliminary clues on their probable biological functions. Compared to the previous works which emphasized the CAMTAs' responses to stresses in other non-crop species, the results of this study will provide some valuable candidates with potential application in crop improvement.

## Materials and methods

### Plant material and treatment conditions

Maize (inbred line “B73”) seeds were treated as the wild-type in our study. After surface sterilized, seeds were soaked in ddH_2_O for 4 h and then germinated in an incubator for another 24 h at 28°C. Seedlings were grown in a growth chamber with a photoperiod of 16/8-h of light/dark and a relative humidity of 60% and light intensity of 120 μmolm^−2^s^−1^. Half-strength modified Hoagland nutrient solution with pH 5.8 was used for seedling growth, with solution changed every 3 days. Two-week-old seedlings were used for hormone and abiotic stress treatment experiments. For hormone treatments, 2-week-old seedlings were transferred to hormone-free nutrient solution or nutrient solution with 10 μM indole-3-acetic acid (IAA), 100 μM ABA, 100 μM SA and 100 μM methyl (Me)-JA for 1 day respectively, and then the root and shoot samples were collected for qRT-PCR experiment. For cold treatment, the seedlings were keeping in a 4°C growth chamber for 24 and 48 h, with untreated seedlings as controls. For drought stress treatment, roots of maize seedlings were soaked in nutrient solution with 10 and 20% polyethylene glycol (PEG) 6000 for 4 days, with untreated seedlings as controls. For salt stress treatment, roots of maize seedlings were soaked in nutrient solution containing 50 and 100 mM NaCl for 4 days, with untreated seedlings as controls. Student's *t*-test analysis between the mock and stress-inoculated plants was used to reveal the differential expression patterns of *ZmCAMTA* family genes.

### Identification and analysis of *CAMTA* gene families in maize

The amino acid sequences of six AtCAMTA proteins were used as queries to BLAST against the maize genome database (http://www.maizegdb.org/) to identify novel maize CAMTA genes. The acceptable e-value in the BLAST analysis for CAMTA identification was set to “-3.” The Hidden Markov Model (HMM) profiles of the *CAMTA* gene family (Pfam 03859: CG-1 DNA-binding domain; Pfam 01833: TIG domain; Pfam 12796: ankyrin repeats; Pfam 00612: IQ motifs) were employed to identify the ZmCAMTAs from the hits (Finn et al., 2014). The amino acid sequences, as well as protein information regarding each matched protein, were obtained. Information concerning these ZmCAMTA proteins was listed in Table S1.

### Phylogenetic relationship, intron-exon structure and genome distribution analysis of *ZmCAMTA* genes

Alignment of maize ZmCAMTA proteins was obtained by the ClustalW program with the default parameters (http://www.ebi.ac.uk/Tools/msa/clustalw2/). The data was visualized by software GeneDoc (http://www.nrbsc.org/gfx/genedoc/), and a phylogenetic tree was built with nine maize, seven rice, six *Arabidopsis*, and 15 soybean CAMTA protein sequences using software MEGA5.1 (http://www.megasoftware.net/) employing the neighbor-joining method. Thousand iterations were used to calculate bootstrap values. The gene pairs displaying high bootstrap value (more than 99%) were identified as sister-pairs. The coding sequences were isolated from the maize database. Exon-intron organizations of *ZmCAMTA* genes were identified by software GSDS 2.0 (http://gsds.cbi.pku.edu.cn/). Circle display of synteny blocks was drawn using the SyMAP database (http://www.symapdb.org/projects/fabaceae/). All *ZmCAMTA* genes were located on 10 chromosomes according to their starting positions. Motif constitution of *ZmCAMTA* proteins was investigated by Multiple Expectation Maximization for Motif Elicitation web server (http://meme.nbcr.net/meme/cgi-bin/meme.cgi).

### Auxin and stress-related *cis*-elements analysis

The promoters (–1500 to −1 bp before the UTRs) of *ZmCAMTA* genes were scanned for the locations of stress related *cis*-acting regulatory elements using Regulatory Sequence Analysis Tools (http://rsat.ulb.ac.be/rsat/). The sequence data of *ZmCAMTA* promoters were got from phytozome 10.1 database. Nine *cis*-elements were used in our study were listed as follow. There are dehydration and cold response (DRE/CRT, RCCGAC), ABA responsive element (ABRE, YACGTGK), SA-responsive promoter element (SARE, TGACG), ARF1 binding site (AuxRE, TGTCTC), environmental signal response (G-box, CACGTG), CAMTA binding site (CG-box, VCGCGB), WRKY binding site (W-box: TTGACY), PHR1 binding site (P1BS, GNATATNC), and sulfur-responsive element (SURE, GAGAC).

### RNA isolation and quantitative RT-PCR

Total RNA was extracted from 50 mg different tissues and organs, such as roots, leaves, shoots, and tassels using RNeasy plant mini kits (Qiagen, Hilden, Germany) following the protocol. Any genomic DNA contamination was removed by DNase I. For each sample, cDNA was synthesized from 3.0 μg total RNA by SuperRT Reverse Transcriptase (CoWin Biotech, Beijing) using oligo (dT) primers. The primers sequences of qRT-PCR were designed and listed in Table S1. The *ZmACTIN* (LOC100284092) gene and *18S rRNA* gene were used as an internal standard to calculate relative expression differences basing on the comparative cycle threshold (2^−Δ*ΔCt*^) values. The internal control genes were used for normalization and the control treatment was used as the calibrator.

The steady-state mRNA levels of *ZmCAMTA* genes were examined by absolute quantification methods in a given tissue or organ. We amplified the partial coding sequence of each *ZmCAMTA* gene from shoot cDNA sample with a high-fidelity polymerase and primers sequences of qRT-PCR (Table S1). The PCR products were separated by agarose gel electrophoresis. DNA product used for standard curves for absolute quantification was purified from Plant DNA Purification Kit (K1830-01, Invitrogen). The purified DNA product was then measured by absorbance at 260 nm (1.0 A_260_ = 50μg/ml).

Copy number of standard stock solutions, presented as the per unit volume, were calculated using the molecular weight of each purified DNA (1 bp = 660 Da) and the Avogadro constant (NA = 6.022 × 10^23^) in the formula below: $transcript\;copies=\frac{NA\;\times transcript\;quantity}{MW}$. These standard stocks were serially diluted to obtain standard series ranging from 10^2^ to 10^9^ copies of amplicon per 1 μl, each step differing by 10-fold. Since a double-stranded PCR product DNA was treated as standard to quantify a single-stranded cDNA of target gene, thus there is no amplification occurring during the first PCR cycle, one cycle must be subtracted from sample *Ct* value. Error related to absolute quantification of each *ZmCAMTA* sample was analyzed as described by Lu et al. (2012).

Several maker genes were used as controls for validating stress conditions. For hormone treatments, *ZmSAUR2* (GRMZM2G156470) was used as a marker gene for IAA treatment; *ZmSNAC1* (GRMZM2G347043) was used as a marker gene for ABA treatment; *ZmJAZ14* (GRMZM2G064775) was used as a marker gene for JA treatment; *ZmLEA3* (GRMZM2G096475) was used as a marker gene for SA treatment. A well characterized abiotic stress inducible marker gene, *ZmDREB1A* (GRMZM2G124037), was used as control for validating abiotic stresses conditions, including salt, drought and cold. For qRT-PCR, 2 μL of a 1/10 dilution of cDNA in water was added to 10 μL of 2 × UltraSYBR (with Rox) (CoWin Biotech, Beijing), 200 nM of each primer and water was then added to make a final volume of 20 μL. The PCR reaction was performed as follows: 95°C for 10 min; 40 cycles of 95°C for 15 s, 60°C for 60 s.

### Test materials and propagation of a viruliferous planthopper population

A susceptible maize inbred “478” and a resistant inbred line “P138” were used as hosts (Huang et al., 2002; Miao et al., 2014). The rice black-streaked dwarf virus (RBSDV)-susceptible wheat cultivar “Shixin 828” was treated as the feeding and reproductive host plants for planthoppers, and for virus maintenance and inoculation source in our experiments. Maize plants of both “478” and “P138” were grown in a greenhouse at nine per row and 81 seedlings per tray for inoculation. Adult planthoppers were collected form “Shixin 828” plants at the edges of an open area in spring by a small sweep net. Insects were moved to wheat seedlings in a 10 × 40 × 20 cm clear glass base sealed with a large cheesecloth. Adult planthoppers on wheat plants were moved to a growth room at 26 ± 2°C with a photoperiod of 14-h light/10-h dark and 70% relative humidity. After 3 weeks, newly hatched first instar nymphs were collected in a container, then moving the nymphs into a cage with fresh wheat seedlings for rearing every week. All adult planthoppers were allowed to lay eggs on “Shixin 828” plants for only 2 days to generate nymphs at the same development stage. RBSDV presence in wheat plants was checked by an indirect enzyme linked immunosorbent assay (Wang et al., 2006).

### RBSDV inoculations

Viruliferous planthoppers were collected by instar nymphs for 5 h and then released at 100 planthoppers per maize plant for a 3-d acquisition period on RBSDV-infected wheat plants. The planthoppers were transferred to healthy wheat seedlings (70 seedlings per pot) for an incubation period of 25 days in a growth chamber with the same condition as above. Trays (70 × 60 × 20 cm) into which a single row of 10 seeds per maize genotype were planted were covered with 40-mesh net cages (70 × 60 × 60 cm) and placed in a shade house until plants were at the 2–3 leaf stage. Then, about 10 viruliferous adult planthoppers per plant were placed onto the maize seedlings in the net cage for a 6-day inoculation access period. Seedlings inoculated with virus-free adult planthoppers under the same conditions were used as control plants.

### qRT-PCR to confirm the RBSDV content in maize materials

After incubation, qRT-PCR was used to confirm the RBSDV contents in different maize materials. Samples from the base of the upper 1–3 young leaves were collected and used for RNA isolation. Three randomly selected samples were collected from inoculated and uninoculated controls of inbred “478” and “P138,” respectively. A pair of RBSDV-specific primers [P1: TCA GCA AAA GGT AAA GGA ACG and P2 (RBSDV): AGA GCT CTT CTA GTT ATT GCG] was designed basing on the sequence of RBSDV S6, which is the major outer capsid proteins. The *ZmACTIN* (LOC100284092) and *18S rRNA* genes were used as an internal standard to calculate relative fold differences in RBSDV proliferation based on the comparative cycle threshold (2^−Δ*ΔCt*^) values. All the expression analysis was carried out for five biological replicates and the values shown in figures represent the average values of these five replicates.

### Construction of protein-green fluorescent protein (GFP) fusion vectors and sub-cellular localization analysis

The coding regions of *ZmCAMTA* cDNAs were cloned into the vector pH7FWG2.0 to generate the protein-GFP expression constructs. An artificial GFP, fused in the frame to the C-terminus of each ZmCAMTA protein, was place under the control of cauliflower mosaic virus (CaMV) 35S promoter. All primer sequences were listed in Table S1. These constructs were used in transient expression in tobacco (*Nicotiana benthamiana*) epidermis cell using *Agrobacterium* transformation. Fluorescence of the fusion protein was detected using a confocal microscope LSM710 (Carl Zeiss, Oberkochen, Germany, http://corporate.zeiss.com/).

### Statistical analysis

Differences between values were calculated using One-Way analysis of ANOVA with Student's *t*-test at a significance level of 0.05 in software Excel. All the expression analysis was performed for five biological repeats and the values shown in figures represent the average values of five repeats, and the data are expressed as the mean and standard deviation (mean ± SD).

## Results

### Identification and structural analyses of *CAMTA* genes in maize

**Figure 1:**
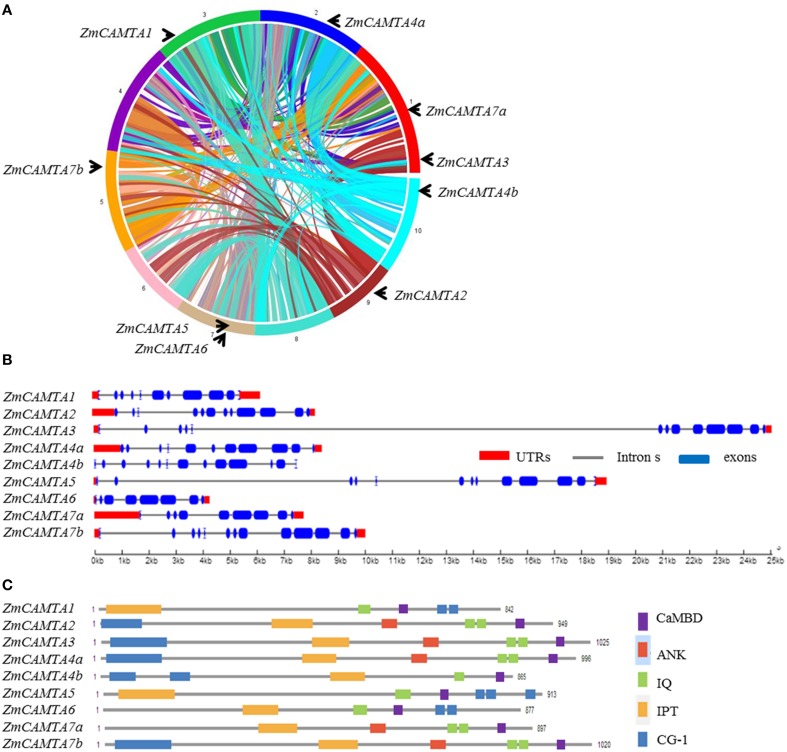
**Chromosomal distribution, protein domain distribution, and gene structural analysis of** ***ZmCAMTA*** **family genes**. **(A)** The genome visualization tool SyMAP Synteny Browser was employed (http://www.symapdb.org/) to analyze the maize genome. Maize chromosomes were arranged in circle. Nine *ZmCAMTA* genes were located in the chromosomes by locus. **(B)** Schematic representation of functional domains of ZmCAMTA proteins. Bioinformatics analysis of the conserved domains was conducted in the Pfam database (http://pfam.janelia.org/). Cam-binding domains (CaMBD) were specifically searched in Calmodulin Target Database (http://calcium.uhnres.utoronto.ca/ctdb/ctdb/). **(C**) Exon-intron structure analysis of *ZmCAMTA* genes. The untranslated regions (UTRs) are indicated by thick red lines; the exons are indicated by blue boxes; the introns are indicated by gray lines.

The protein sequences of six *Arabidopsis* CAMTAs were used to identify homologs of CAMTA in maize by BLAST in the Phytozome 10.1 database. A total of nine *ZmCAMTA* genes were identified and their protein sequences of these genes were downloaded and confirmed by the HMM profiles of the CAMTA family (Pfam 03859: CG-1 DNA-binding domain; Pfam 01833: TIG domain; Pfam 12796: ankyrin repeats; Pfam 00612: IQ motifs). These genes were named according to their locations on the chromosomes (from Chr. 1 to Chr. 10). All *ZmCAMTA* genes were mapped on seven chromosomes unevenly. Chromosomes 1 and 7 contained two *ZmCAMTA* genes (*ZmCAMTA2* and *ZmCAMTA7a* on chromosome 1 and *ZmCAMTA5* and *ZmCAMTA6* on chromosome 7). Chromosomes 2, 3, 5, 9, and 10 contained one *ZmCAMTA* gene each (Figure 1A).

Comprehensive information on *ZmCAMTA* genes, including gene names, locus ID, open reading frame (ORF) length, intron-exon number, location on chromosome and basic parameter of deduced polypeptide, were listed in Table 1. The sizes of the deduced ZmCAMTA proteins greatly varied from 842 (ZmCAMTA1) to 1025 amino acids (ZmCAMTA3), the corresponding molecular mass varied within the range of 94.65–114.41 kDa, and the predicted isoelectric point varied widely from 5.19 (ZmCAMTA7a) to 8.25 (ZmCAMTA5). The average genomic DNA length of *ZmCAMTA* genes was approximately 10.72 kb, which was much longer than *Arabidopsis* (about 5.3 kb). Interestingly, a large number of introns were contained in *ZmCAMTA* genes (from 7 to 12) (Table 1). Most *ZmCAMTA* genes showed a similar exon-intron structural pattern, indicating a necessary conservation in genomic structure of *ZmCAMTA* genes (Figure 1B). All ZmCAMTAs contained a highly conserved domain structure (Figure 1C), and the sequence alignments results were showed in Figure S1.

**Table 1:** ***CAMTA*** **family genes in maize**.

**Gene name**	**Locus ID**	**ORF length (bp)**	**No. of introns**	**Chr No**.	**Deduced polypeptide**		
					**Length (aa)**	**Mol wt (kDa)**	**pI**
ZmCAMTA1	GRMZM2G171600	2529	10	3	842	94.65	6.75
ZmCAMTA2	GRMZM2G431243	2850	12	9	949	106.16	6.36
ZmCAMTA3	GRMZM2G447551	3078	12	1	1025	114.41	6.22
ZmCAMTA4a	GRMZM2G143205	2991	11	2	996	112.2	6.36
ZmCAMTA4b	GRMZM2G152661	2598	12	10	865	97.52	7.58
ZmCAMTA5	GRMZM2G032336	2742	12	7	913	101.84	8.29
ZmCAMTA6	GRMZM2G017368	2634	7	7	877	96.8	5.21
ZmCAMTA7a	GRMZM2G153594	2694	10	1	897	98.61	5.19
ZmCAMTA7b	GRMZM2G341747	3063	12	5	1020	113.25	5.59

To confirm the nucleic localization of ZmCAMTAs, we observed transient expression of ZmCAMTA: GFP fusion proteins in epidermal cells of *N. benthamiana* leaves. Our data showed that all the ZmCAMTA proteins are localized in nucleus (Figure S2).

### Phylogenetic analysis of *CAMTA* genes

**Figure 2:**
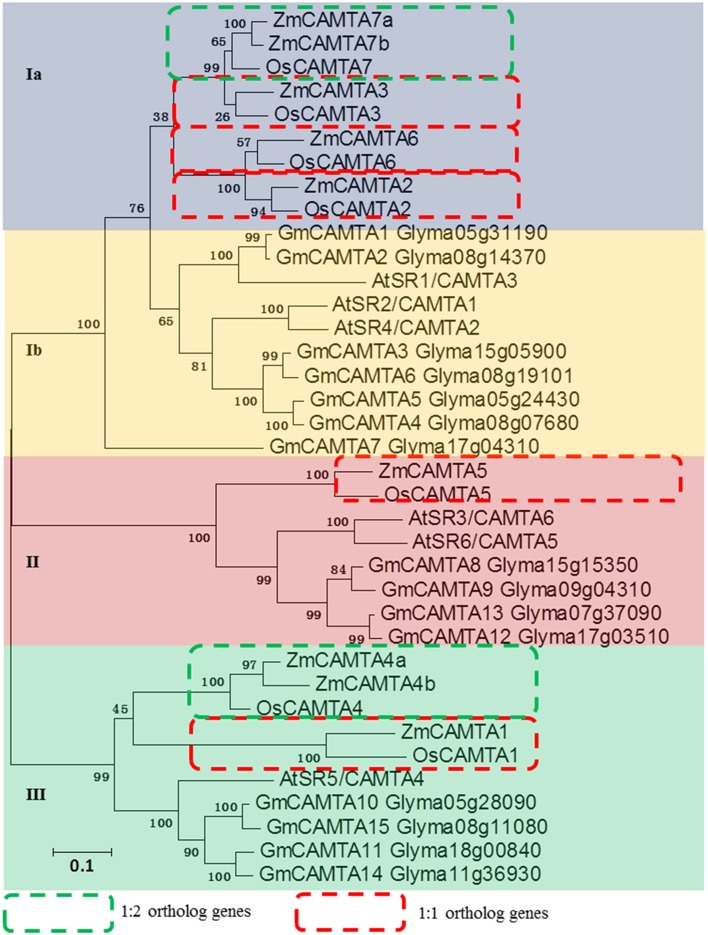
**Phylogenetic relationship analysis of** ***CAMTA*** **gene families between maize,** ***Arabidopsis***, **rice, and soybean**. Seven rice CAMTAs, 15 soybean CAMTAs, six *Arabidopsis* CAMTAs and nine maize CAMTAs were used to build this phylogenetic tree with NJ method. Amino acid sequences of these different plants CAMTA proteins were used for analysis. Bootstrap values are presented for all branches. Different colors indicated different subfamilies (I, II, and III). The 1:1 ortholog genes between maize and rice were indicated by red dotted boxes. The 1:2 ortholog genes between maize and rice were indicated by green dotted boxes.

Many studies have revealed the biological functions of *CAMTA* family genes in the model plant *Arabidopsis*, monocotyledonous rice and leguminous soybean (Bouche et al., 2002; Koo et al., 2009; Wang et al., 2015). In the present study, two phylogenetic trees were built with different methods to analyze the relationships of *CAMTA* genes among *Arabidopsis*, soybean, rice, and maize. The data showed that all 37 *CAMTA* genes were grouped into four subfamilies (from Ia to III). Four 1:1 ortholog gene-pairs with high bootstrap value (more than 99%) were found between maize and rice: *ZmCAMTA3*/*OsCAMTA3, ZmCAMTA6*/*OsCAMTA6, ZmCAMTA1/OsCAMTA1*, and *ZmCAMTA5*/*OsCAMTA5*. Furthermore, two 2:1 ortholog gene pairs with more than 99% bootstrap value were identified between maize and rice: *ZmCAMTA7a*/*ZmCAMTA7b/OsCAMTA7* and *ZmCAMTA4a*/*ZmCAMTA4b/OsCAMTA4* (Figure 2 and Figure S3). No ortholog gene-pair of *CAMTA* was identified between soybean and maize. Notably, the subfamily Ia was monocot-specific subfamily, and no *ZmCAMTA* genes belonged to subfamily Ib. Furthermore, a separate phylogenetic tree with the sequences of ZmCAMTA proteins was built to determine phylogenetic relationship of *CAMTA* genes in maize (Figure S4).

### Tissue-specific expression patterns of *ZmCAMTA* genes

Determination of the tissue-specific expression patterns of *ZmCAMTA* genes provided us new insights into their roles in different organs of maize. In our study, absolute expression levels of *ZmCAMTA* genes in the roots (R), leaves (L), shoots (S) of 2-week-old hydroponic maize seedlings and tassels (F) of 2-month-old plants were analyzed by qRT-PCR. The standard curve of each *ZmCAMTA* gene was calculated and showed in Figure S5.

**Figure 3:**
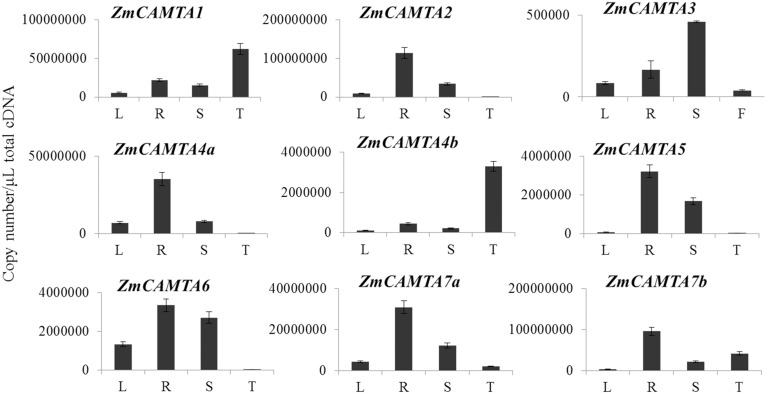
**Tissues-specific expressions of** ***CAMTA*** **family genes in maize**. Quantitation of absolute copy numbers of *ZmCAMTA* family genes in four indicated organs were analyzed by qRT-PCR data. RNA samples were extracted from the shoot (S), roots (S), and leaves (L) of 2-week seedling and the tassels (T) of 2-month plants. The data were analyzed by five independent repeats, and standard deviations were shown with error bars.

Quantitation of absolute copy numbers of *ZmCAMTA* family genes were detected in all tissues and organs. Most *ZmCAMTA* genes, including *ZmCAMTA2, ZmCAMTA4a, ZmCAMTA5, ZmCATMA6, ZmCAMTA7a*, and *ZmCAMTA7b*, showed higher expression levels in roots than other organs. The expression of *ZmCAMTA1* was lower in leaves and shoots than that in roots and tassels. The transcript levels of *ZmCAMTA2, ZmCAMTA4a, ZmCAMTA5*, and *ZmCAMTA6* were hardly detectable in tassels, indicating that they had a limited or no role in flowering and reproduction. *ZmCAMTA1* and *ZmCAMTA4b* showed tassel-specific expression. Only ZmCAMTA displayed shoot-specific expression (Figure 3).

### *Cis*-acting regulatory elements in the promoter regions of *ZmCAMTA* family genes

Several *cis*-elements that involved in stress responses have been well-identified in the model plants. There were DRE/CRT (Sakuma et al., 2002), ABRE (Osakabe et al., 2014), AuxRE (Ulmasov et al., 1997), SARE (Pieterse and Van Loon, 2004), G-box (Williams et al., 1992), W-box (Chen et al., 2012), CG-box (Yang and Poovaiah, 2002), P1BS (Rubio et al., 2001), and SURE (Maruyama-Nakashita et al., 2005).

**Figure 4:**
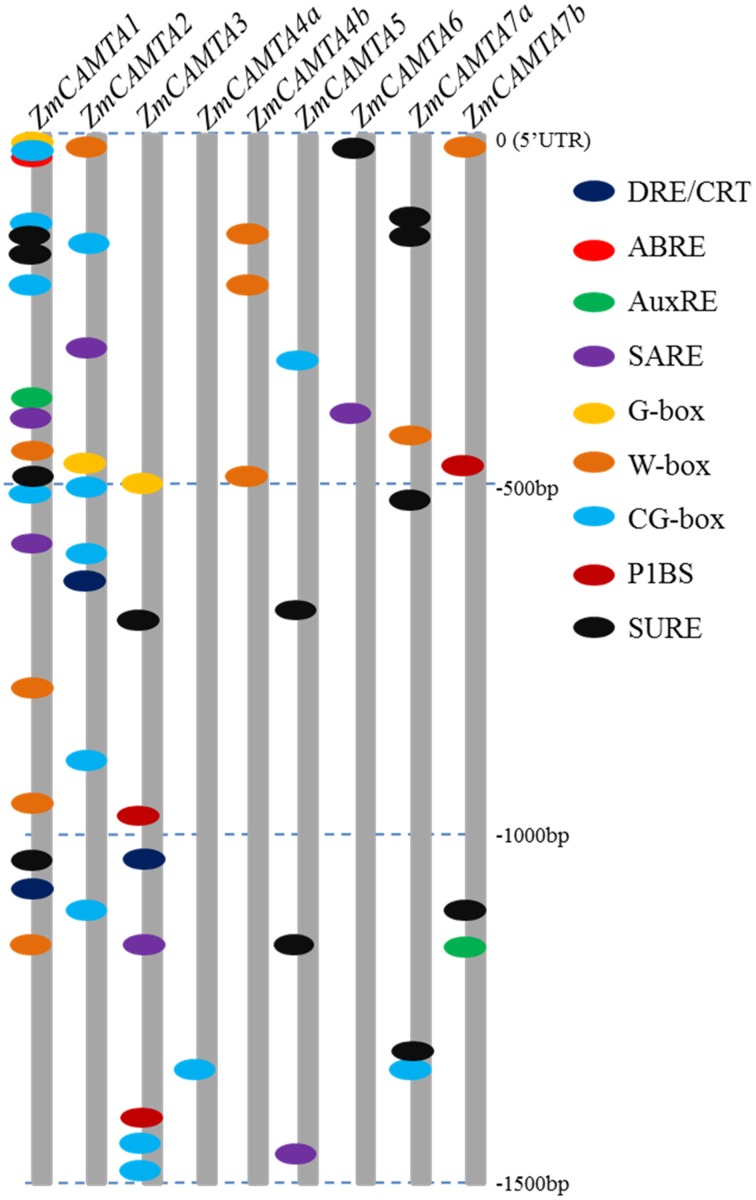
**Motif analyses of stress-related** ***cis*****-element in promoters of** ***ZmCAMTA*** **family genes**. The 1500 bp promoter regions of corresponding *ZmCAMTA* genes were used to analysis of stress-related cis-elements, which were given using the presented color code. Nine *cis*-elements were used in this study: dehydration and cold response (DRE/CRT), ABA responsive element (ABRE), ARF1 binding site (AuxRE), SA-responsive promoter element (SARE), environmental signal response (G-box), WRKY binding site (W-box), CAMTA binding site (CG-box), PHR1 binding site (P1BS) and sulfur-responsive element (SURE). The sequences of these *cis*-elements were showed in the right of figure.

The 1500 bp upstream of *ZmCAMTA* promoters were obtained from Phytozome and nine stress-related *cis*-elements were scanned for clues on how the expression levels of *ZmCAMTA* genes responded to stress stimuli. Some stress-related motifs were contained in the promoters of *ZmCAMTA* genes (Figure 4). The data showed that several stress-related *cis*-elements, such as SARE, W-box, CG-box, and SURE, were enriched in the promoters of *ZmCAMTA* genes. In total, 10 W-box, 16 CG-box, and 13 SURE *cis*-elements were included in the *ZmCAMTA* promoters. The numbers of stress-related *cis*-elements in the upstream 1.5 kb regions of *ZmCAMTA* family genes were summarized in Table S2.

### Expression levels of *ZmCAMTA* genes in response to phytohormone stimuli

Stress related hormones (e.g., IAA, SA, ABA, and JA) have been well studied for their participation in plant growth and development (Xiong et al., 2002). To understand how *ZmCAMTA* genes were participated in stress-related hormone responses, qRT-PCR was used to analyze the expression of *ZmCAMTA*s under 10 μM IAA, 100 μM ABA, 100 μM SA, and 100 μM Me-JA in the leaves and roots for 12 h, respectively. Five well-characterized marker genes (auxin-inducible *ZmSAUR*, ABA-inducible *ZmSNC1*, JA-inducible *ZmJAZ4*, SA-inducible *ZmLEA3*, and abiotic stress-inducible *ZmDREB1A*) were used as controls for validating stress and hormone conditions. Our data showed that all the stress-inducible marker genes were greatly induced by the stress treatments (Figure S6).

**Figure 5:**
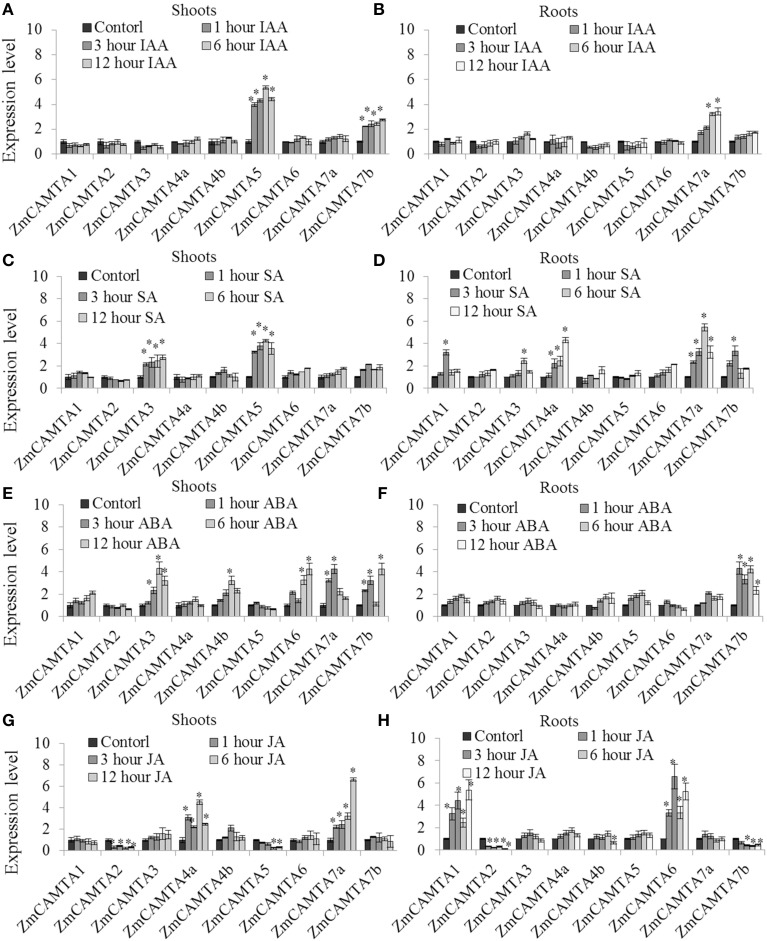
**Expression of nine** ***ZmCAMTA*** **genes in responses to IAA, SA, ABA, and JA treatments**. Expression of *ZmCAMTA* genes were analyzed by qRT-PCR in both the shoots and roots of 2-week-old maize seedlings. The expression levels of *ZmCAMTA* genes in control seedlings were normalized to a value of 1. **(A,B)** The expression levels of *ZmCAMTA* genes in 10 μM IAA treated seedlings were compared to control treatments as relative mRNA levels. **(C,D)** The expression levels of *ZmCAMTA* genes in 100 μM SA treated seedlings were compared to control treatments as relative mRNA levels. **(E,F)** The expression levels of *ZmCAMTA* genes in 100 μM ABA treated seedlings were compared to control treatments as relative mRNA levels. **(G,H)** The expression levels of *ZmCAMTA* genes in 100 μM Me-JA treated seedlings were compared to control treatments as relative mRNA levels. Error bars represent standard deviations from five biological replicates. A specific fold change value (2x) in the expression levels is used to clarify the statistical analysis of significant differences among mock and the treatments. The significant differences were indicated by an asterisk.

The relative expression levels of *ZmCAMTA* genes under IAA, SA, ABA, and JA treatments compared to mock treatments were showed in Figure 5. The expression of *ZmCAMTA5* and *ZmCAMTA7b* was significantly up-regulated by IAA treatment in the leaves (Figure 5A), and during the 12 h periods, the expression of *ZmCAMTA5* and *ZmCAMTA7b* reached a maximum at 1 h after IAA treatment; while the expression level of *ZmCAMTA7a* was significantly induced by IAA treatment in the roots (Figure 5B). The expression levels of *ZmCAMTA3* and *ZmCAMTA5* were significantly induced by SA treatment in leaves (Figure 5C), and expression levels of *ZmCAMTA2* and *ZmCAMTA3* were significantly induced by SA treatment in the roots (Figure 5D). Under ABA treatment, *ZmCAMTA3, ZmCAMTA4b, ZmCAMTA6, ZmCAMTA7a*, and *ZmCAMTA7b* were induced in the shoots and only *ZmCAMTA7b* was induced in the roots (Figures 5E,F). *ZmCAMTA4a* and *ZmCAMTA7a* were largely up-regulated by Me-JA treatment in the shoots, and another two genes, *ZmCAMTA1* and *ZmCAMTA6* were obviously induced by Me-JA treatment in the roots (Figures 5G,H). Thus hormone signal molecules involved in plant responses to environmental stresses could also regulate expression of *ZmCAMTA* genes.

### Expression of *ZmCAMTA* genes in responses to cold, drought and salt treatments

Cold, drought, and high salinity are major abiotic stresses frequently experienced by maize plants under various natural conditions (Xia et al., 2012; Wang et al., 2015). Ca^2+^ signaling-related gene transcriptional regulation is an important process required for crops to survive and adapt to adverse environmental stresses (Pardo et al., 1998; Magnan et al., 2008; Choi et al., 2014). In the present study, the expression patterns of *ZmCAMTA* genes under cold, NaCl, and PEG treatments were analyzed to investigate their potential roles in maize tolerance to abiotic stresses. The data of phenotypic alterations were showed as Figures S7–S9.

**Figure 6:**
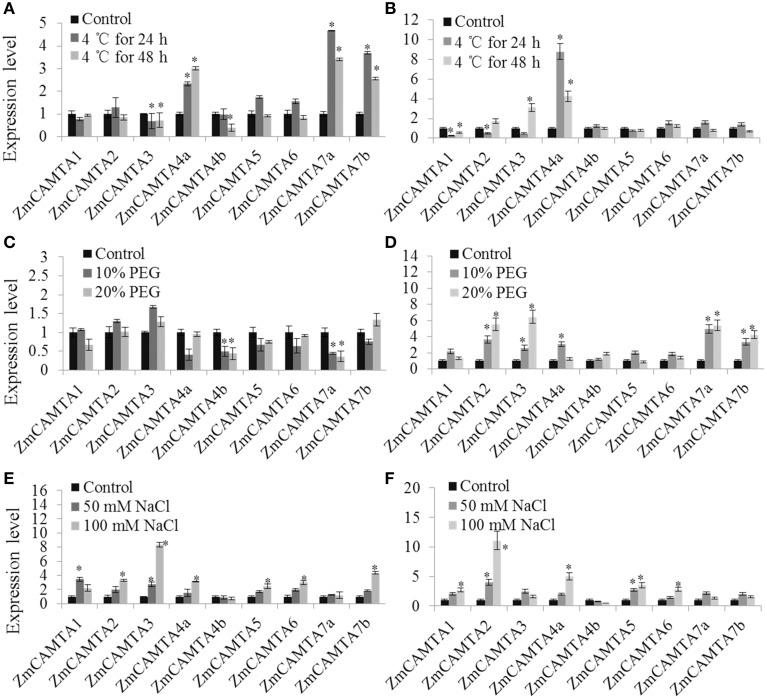
**Expression of nine** ***ZmCAMTA*** **genes in response to Cd, PEG, and NaCl treatments**. Expression of *ZmCAMTA* genes were analyzed by qRT-PCR in both the shoots and roots of 2-week-old maize seedlings. The expression levels of *ZmCAMTA* genes in control seedlings were normalized to a value of 1. **(A,B)** The expression levels of *ZmCAMTA* genes in 4°C treated seedlings were compared to control treatments as relative mRNA levels. **(C,D)** The expression levels of ZmCAMTA genes in PEG treated seedlings were compared to control treatments as relative mRNA levels. **(E,F)** The expression levels of ZmCAMTA genes in NaCl treated seedlings were compared to control treatments as relative mRNA levels. Error bars represent standard deviations from five biological replicates. The significant differences among control and the treatments were indicated by an asterisk.

In the cold treatment, *ZmCAMTA4a, ZmCAMTA7a*, and *ZmCAMTA7b* were significantly up-regulated in the shoots, and only *ZmCAMTA4a* was largely induced in the roots; the expression levels of *ZmCAMTA3* and *ZmCAMTA4b* were reduced by cold treatment in the shoots (Figures 6A,B). There were no significant changes in expression of most *ZmCAMTA* genes except for *ZmCAMTA4b* and *ZmCAMTA7a* in the PEG-treated shoots; however, *ZmCAMTA2, ZmCAMTA3, ZmCAMTA7a*, and *ZmCAMTA7b* responded to PEG treatment and their expression reached a peak under 20% PEG treatment (Figures 6C,D). With the exception of *ZmCAMTA4b* and *ZmCAMTA7a*, NaCl treatment significantly increased the expressions of *ZmCAMTA* genes both in the shoots and roots. The expression levels of *ZmCAMTA* genes under 100 mM NaCl treatment were higher than that 50 mM NaCl treatment (Figures 6E,F). Thus, *ZmCAMTA* genes were transcriptionally responsive to abiotic stresses of cold, salt, and drought.

### Expression responses to RBSDV infection

Maize rough dwarf disease (MRDD) is a devastating viral disease of crops worldwide (Tao et al., 2013). The RBSDV is the major cause of MRDD in East Asia, and was used for the virus infection experiment.

To confirm the presence of RBSDV in the tested maize plants, RBSDV-specific qRT-PCR was used to calculate the virus proliferation rates. The data showed that the RBSDV was present in both maize plants. After RBSDV infection, the virus proliferation rate in the susceptible maize “478” was much higher than that in the resistant “P138” (Figure S10). The differences in virus proliferation rate between “478” and “P138” was in agreement with the differences in the disease resistant phenotype reported previously (Huang et al., 2002; Miao et al., 2014).

**Figure 7:**
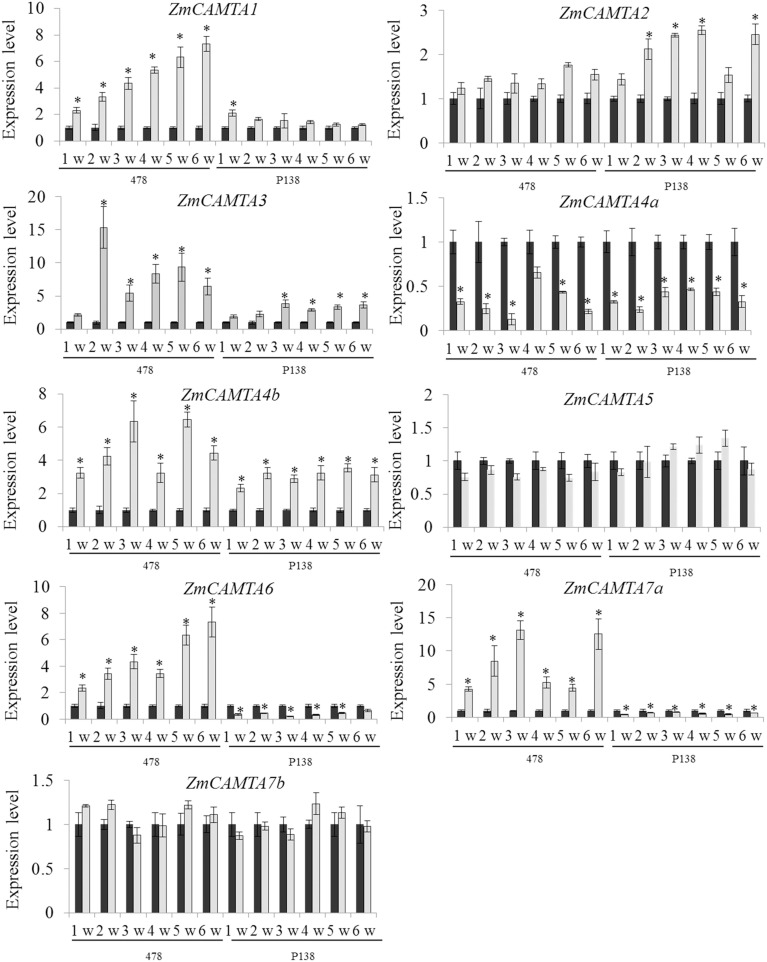
***ZmCAMTA*** **genes expression pattern at the early phase of** ***rice black-streaked dwarf virus*** **infection**. A susceptible maize inbred “478” and a resistant inbred line “P138” were used to test the changes of *ZmCAMTA* genes expression level at different time points (1, 2, 3, 4, 5, and 6 week) and their control treatments. In our experiment, significant differences among control and the infection treatments were indicated by *P < 0.05.

In our study, the expression patterns of *ZmCAMTA* genes under RBSDV were analyzed to investigate their potential roles in response of different varieties to RBSDV over a 6-week period as described in the Materials and Methods. Five *ZmCAMTA* genes, including *ZmCAMTA1, ZmCAMTA3, ZmCAMTA4b, ZmCAMTA6*, and *ZmCAMTA7a*, were significantly up-regulated and *ZmCAMTA4a* was down-regulated by RBSDV infection in the susceptible “478.” However, only *ZmCAMTA3* and *ZmCAMTA4b* significantly increased and *ZmCAMTA4a, ZmCAMTA6*, and *ZmCAMTA7a* decreased under RBSDV infection in resistant “P138.” Furthermore, seven sampling time points (0, 1, 2, 3, 4, 5, and 6 weeks) were used to test whether *CAMTA* genes in maize were RBSDV infection early response genes. In the susceptible maize inbred “478,” most *ZmCAMTA* genes showed quick responses to virus infection. Expression of *ZmCAMTA3* reached a maximum at 2 week after virus infection, and then slightly decreased. Expression of *ZmCAMTA4a* reached a minimum at 3 week after virus infection, and then slowly recovered. The expression of *ZmCAMTA1* and *ZmCAMTA6* increased gradually over the 6 week-period after infection. *ZmCAMTA5* and *ZmCAMTA7b* showed no significantly changes in the 6 week-period after virus infection. In the resistant “P138,” the expression of *ZmCAMTA4a* reached a minimum at 3 week after virus infection. The expression of *ZmCAMTA3* reached a maximum at 3 week after virus infection, and then slightly decreased. These results confirmed that *ZmCAMTA* genes were transcriptionally responsive to RBSDV infection (Figure 7).

## Discussion

“Ca^2+^ signatures,” the specific changes in intracellular Ca^2+^ concentration, have been reported to be involved in plants adapting to changing environments (McAinsh and Pittman, 2009). A large number of Ca^2+^-binding proteins as well as their downstream target proteins regulated by the Ca^2+^ sensors comprise the complex Ca^2+^ signaling pathway (Poovaiah and Reddy, 1987; Poovaiah et al., 2013). As a classical Ca^2+^-binding protein found in all eukaryotes, CaM has roles in biochemistry, cell biology, and molecular biology due to its importance in almost all aspects of cellular regulation (Bouche et al., 2005; Du et al., 2009; Defalco et al., 2010; Reddy et al., 2011). Many TFs, including CAMTA, WRKY, MYB, and bZIP, have been shown to be CaM-binding proteins by interacting with CaMs/CMLs and responding to different abiotic and biotic signal stresses (Yang and Poovaiah, 2002; Finkler et al., 2007; Yang et al., 2013). CAMTA-mediated gene transcription regulation is a key process for plants responses to exogenous hormones and abiotic stresses (Galon et al., 2008; Doherty et al., 2009; Du et al., 2009; Nie et al., 2012; Qiu et al., 2012). In our study, a comprehensive analysis of maize *CAMTA* family genes and their expression patterns under various abiotic and biotic stresses was performed to identify candidates involved in abiotic responses. Systematic characterization of these transcriptional mediators provides clues to understand the mechanisms by which Ca^2+^-signaling triggers appropriate environmental responses in a timely and tissue-specific manner.

Nine members of maize *CAMTA* gene family were identified. The number of *ZmCAMTA* genes is more than for *Arabidopsis* (six members) and is less than for soybean (15 members) (Schmutz et al., 2010; Wang et al., 2015). A high degree similarity of sequences and structural patterns of ZmCAMTA proteins indicates that these *ZmCAMTA* genes may originate from one ancestral sequence (Figure S1). A complete phylogenetic tree of rice, *Arabidopsis*, soybean and maize was built to analyze the relationships of CAMTAs among these four species. *CAMTA* genes in maize were found to have homologs in rice. Whole genome duplication is predicted to occur in the ancestor of monocots about 70 million years ago, before the divergence of maize and rice (Paterson et al., 2004). Based on the phylogenetic analysis, six sister-pairs between maize and rice were identified as ortholog genes with bootstrap value =99%, suggesting that the functions of these ZmCAMTAs might be similar to the CAMTAs in rice. No sister-pair genes were identified between maize and soybean (Figure 2). Divergence in evolution of CAMTA family members across various plant species may exist (Wang et al., 2015). Furthermore, a separate phylogenetic tree was built with all the CAMTA protein sequences from maize (Figure S4). ZmCAMTAs in the same subfamily always showed different gene structure and protein domain composition, suggesting a diversity of bio-functions within the phylogenetic subfamilies.

Spatial differences in plant CAMTA gene expressions were previously implicated in growth and development. *Arabidopsis AVP1*, a H^+^-pyrophosphatase encoding gene, was identified as a downstream target of CAMTAs (Mitsuda et al., 2003). AtCAMTA1 as well as AtCAMTA5 possibly enhance pollen-specific expression of *AVP1* during pollen development (Li et al., 2005). Expression of *NtER1*, a tobacco *CAMTA* homologous gene, shows high levels in senescing leaves and flower petals, implying that NtER1 is developmentally regulated and acts as a trigger for senescence and death (Yang and Poovaiah, 2000). Some *CAMTA* genes in tomato showed strong expression in the fruit, suggesting a close relationship between their potential roles and fruit development and ripening (Yang et al., 2012). The spatio-temporal expression pattern indicated that most *ZmCAMTA* genes were highly expressed in the roots. How *ZmCAMTA* genes play a putative role in the root system architectures under various environmental stimuli needs further investigation.

Environmental stresses cause changes in gene expression (Schutzendubel and Polle, 2002; Atkinson et al., 2013). *CAMTAs* in different species are reported to be responsive to diverse environmental stresses, such as high salinity, drought, and heavy metal toxicity (Doherty et al., 2009; Pandey et al., 2013; Yang et al., 2013). Moreover, *CAMTA* genes are also involved in the crosstalk between stresses and stress-related hormones (Reddy et al., 2000; Yang and Poovaiah, 2002). *Cis*-elements analysis suggested that several stress-related motifs are contained in the promoter of *ZmCAMTA* genes (Figure 4). Notably, many stress-related elements present in the promoter regions of *ZmCAMTA1, ZmCAMTA2*, and *ZmCAMTA3*, indicating a genetic basis of stress expression regulation of these genes.

In *Arabidopsis*, CAMTA1 participates in auxin signaling and responds to stresses (Galon et al., 2010b). The expression pattern of *AtCAMTA1* displays significant differences on exposure to increasing salt concentrations, suggesting important evidence for the involvement of *CAMTAs* in salt stress response (Galon et al., 2010a). Other *AtCAMTA* genes, such *AtCAMTA1, AtCAMTA2*, and *AtCAMTA3*, establish roles in freezing tolerance of *Arabidopsis* by inhibiting SA biosynthesis at warm temperature (Doherty et al., 2009; Kim et al., 2013). SA treatment could also increase the expression of *ZmCAMTA4a*, suggesting that *ZmCAMTA4a* may be a key regulator in increasing tolerance of maize to freezing. In rice, *OsCAMTA1* (*LOC_Os01g69910*) was one of the candidate genes in a cold tolerance QTL (qSCT1), and played an important role in cold responses (Kim et al., 2014). In maize, the expression of *ZmCAMTA1*, a homologous gene of *OsCAMTA1*, was significantly reduced by cold treatment in the roots, suggesting that the CAMTA-mediate cold tolerance may exist in different monocots (Figure 6B). Interestingly, *ZmCAMTA4a* was the only one that was significantly reduced by RBSDV infection. Responses of *ZmCAMTA4a* to both SA and virus infection suggested that *ZmCAMTA4a* may trigger an SA-dependent plant immunological network (Galon et al., 2008; Du et al., 2009). Recently, AtCAMTA1 was reported to be involved in drought responses by regulating expression of the *AP2-EREBP* gene and ABA responses. The knockout *camta1* shows drought sensitivity, poor root growth, and decline water use efficiency (Pandey et al., 2013). As potential candidate genes, overexpression of *ZmCAMTA* genes may enhance maize drought tolerance. DRE/CRT-binding factor (CBF) has been identified as the core TF participating in gene regulation under osmotic stress (Shangguan et al., 2014). Seven conserved DNA motifs (from CM1 to CM7) are present in the promoters of *CBF2*, which is induced rapidly in response to low temperature (Doherty et al., 2009; Eckardt, 2009). The CM2 sequence matched the CG-1 consensus sequence for CAMTA proteins, and CAMTA proteins are capable of specific binding to this element (Eckardt, 2009). Interestingly, *ZmCAMTA2* and *ZmCAMTA3* contained DRE/CRT elements in their promoter regions (Figure 4), indicating a feedback regulation of CAMTAs by DREB/CBF transcription factors in maize. In addition, five *ZmCAMTA* genes were responsive to ABA treatment in the shoots but only *ZmCAMTA7b* was responsive to ABA treatment in the roots (Figure 5), suggesting an important role in the signal transduction of plant response to osmotic and cold stress by both ABA-dependent and ABA-independent pathways (Yamaguchi-Shinozaki and Shinozaki, 2006). Our data indicated that *ZmCAMTA2* and *ZmCAMTA3* have potential application in molecular breeding to improve crop cold tolerance.

Recently, a CAMTA from *Arabidopsis* was also reported to participate in plant responses to biotic stresses caused by pathogens and insect bites. A loss-of-function mutant of *AtCAMTA3/SR1* displays a pathogen-resistant phenotype and expression regulation of pathogenesis-related genes (Galon et al., 2008; Du et al., 2009). High levels of endogenous SA enhances plant defense responses, and AtCAMTA3 is reported to be a negative regulator of the SA signaling pathway (Nie et al., 2012). Recently, *AtCAMTA3* was also found to be involved in resistance to insect attack by regulating glucosinolate metabolism (Laluk et al., 2012). The expression of *ZmCAMTA* family genes showed quick responses to RBSDV infection, suggesting that *ZmCAMTA* genes may function to cope with biotic stresses in maize. In particularly, qRT-PCR data suggested that expression differences of *ZmCAMTA6* and *ZmCAMTA7a* genes between “478” and “P138” may be involved in disease resistance in maize plants. In rice, *OsCBT* (*OsCAMTA5*), a *CAMTA* family gene, functions as a negative controller in pathogen defense (Koo et al., 2009; Qiu et al., 2012). However, its homologous gene in maize, *ZmCAMTA5*, showed no responses to RBSDV infection in both “478” and “P138.” It suggested that diverse mechanisms were involved in pathogen defense between rice and maize.

In conclusion, the present study provided comprehensive information about domain structure, exon-intron structure, *cis*-elements, the phylogenetic tree and expression analysis of *CAMTA* genes in maize. The responsiveness of *ZmCAMTA* genes to a wide range of abiotic and biotic treatments suggested that they are involved in the tolerance of maize to environmental stresses. Further studies are needed to advance the understanding of the functions of *ZmCAMTA* genes in maize, an important crop with a complex genome and few mutants.

### Conflict of interest statement

The authors declare that the research was conducted in the absence of any commercial or financial relationships that could be construed as a potential conflict of interest.
