# Supplementary material for: Identification and expression profiling analysis of calmodulin-binding transcription activator genes in maize (Zea mays L.) under abiotic and biotic stresses
Source: Front Plant Sci. 2015 Jul 28;6:576. doi: 10.3389/fpls.2015.00576 (PMC4516887; doi:10.3389/fpls.2015.00576)
Supplement: Supplementary file 1 [file Table1.DOCX]

| Table S1 The primer sequences | |  |
| --- | --- | --- |
| Gene name | UP | Dn |
| ZmCAMTA1-RT | AGTGAAGACCCGCTGGCTAA | CCGCCTCTGAAATGTTGGAT |
| ZmCAMTA2-RT | TGGCTGTTGGAAGAGGATTT | TGGCTGTTGGAAGAGGATTT |
| ZmCAMTA3-RT | CCCCAGAACCACCAAATAGA | TGATTTACCGCCCTTGACTT |
| ZmCAMTA4a-RT | GATTCTTTGGCGGCAGTGAG | TTTGCGACCTTTCCATCCTT |
| ZmCAMTA4b-RT | GTTTCACAGACATCGCACCA | ATCCGTTCCATTGAATCCTC |
| ZmCAMTA5-RT | CCACTGAGGGATTGCTTGAA | GCCAGATCACCAGCAGTTTG |
| ZmCAMTA6-RT | TCCGTGATTCTGAGACCCAT | ACCTTGTCCTTCCTTGCCTA |
| ZmCAMTA7a-RT | TTTGGTCTGTTGGCATCGTG | GTCCATCCCTGCTGACTCCT |
| ZmCAMTA7b-RT | GAGAAAGCAAGCGGCTCAAT | CTCCACGATCCCAACAGACC |
| ZmSAUR2-RT | CACGGTTCGAGGTGCCGC | TGTGTGGCTCCAACATAC |
| ZmSNAC1-RT | CAACGGGTACTGGAAGGCC | GCCGGCGACGGCGGTCTTC |
| ZmJAZ14-RT | CAGGAGGCCAAGGCCACCCT | CTGTCCTTCCTCTTCCTAAG |
| ZmLEA3-RT | CAAGGGCCAGGACGCCAAGG | TCAGTGGCCTGCTGGATGAC |
| ZmDREB1A-RT | GACGAAGCGACCTCGGGCG | TCAGCGGCTCCGGAGTCAC |
| ZmCAMTA1-GFP | ATGGCGGAGGCGCGGCGCCACG | TGACCAAGAAGGCATTGGTA |
| ZmCAMTA2-GFP | ATGTTGGAAGAGGACTATAT | AATATAACCAGGTATAGGTG |
| ZmCAMTA3-GFP | ATGAGCCAGAGTTTTGACAT | CATGAAATTGTTGATCCCT |
| ZmCAMTA4-GFP | ATGCAGCAGCAGCAGCAAGG | CTTCTCCTGATCATCCTTTG |
| ZmCAMTA5-GFP | ATGGCCTCGGCGGAGGCGCGC | AAAATAACCCGGGATAGGTGC |
| ZmCAMTA6-GFP | ATGGCGGGTGGCGCCGGCGGGC | CTTTCGACCGAACTCCACCTT |
| ZmCAMTA7-GFP | ATGGATGCTGAAAGCCCAAT | AGAAATAGTCGACATCGGGGCA |
| ZmCAMTA8-GFP | ATGCCGAGGAGTGGCTCTCTT | TCTGATGTAAAAAGGCTCAGC |
| ZmCAMTA9-GFP | ATGGGGTTTAATAGAATATTAA | CTTCATTTGATCCTTCCAAT |
|  |  |  |
