# Supplementary material for: Identification and expression profiling analysis of calmodulin-binding transcription activator genes in maize (Zea mays L.) under abiotic and biotic stresses
Source: Front Plant Sci. 2015 Jul 28;6:576. doi: 10.3389/fpls.2015.00576 (PMC4516887; doi:10.3389/fpls.2015.00576)
Supplement: Supplementary file 2 [file Table2.DOCX]

| Table S2. Numbers of stress-related cis-elements in the upstream 1.5 kb regions of *ZmCAMTA* family genes | | | | | | | | | |
| --- | --- | --- | --- | --- | --- | --- | --- | --- | --- |
|  | DRE/CRT | ABRE | AuxRE | SARE | G-box | W-box | CG-box | P1BS | SURE |
| ZmCAMTA1 | 1 | 1 | 1 | 2 | 1 | 4 | 4 | 0 | 4 |
| ZmCAMTA2 | 1 | 0 | 0 | 1 | 1 | 1 | 5 | 0 | 0 |
| ZmCAMTA3 | 1 | 0 | 0 | 1 | 1 | 0 | 4 | 2 | 1 |
| ZmCAMTA4a | 0 | 0 | 0 | 0 | 0 | 0 | 1 | 0 | 0 |
| ZmCAMTA4b | 0 | 0 | 0 | 0 | 0 | 3 | 0 | 0 | 0 |
| ZmCAMTA5 | 0 | 0 | 0 | 1 | 0 | 0 | 1 | 0 | 2 |
| ZmCAMTA6 | 0 | 0 | 0 | 1 | 0 | 0 | 0 | 0 | 1 |
| ZmCAMTA7a | 0 | 0 | 1 | 0 | 0 | 1 | 0 | 1 | 1 |
| ZmCAMTA7b | 0 | 0 | 0 | 0 | 0 | 1 | 1 | 0 | 4 |
|  |  |  |  |  |  |  |  |  |  |
