# Supplementary material for: Identification and expression profiling analysis of calmodulin-binding transcription activator genes in maize (Zea mays L.) under abiotic and biotic stresses
Source: Front Plant Sci. 2015 Jul 28;6:576. doi: 10.3389/fpls.2015.00576 (PMC4516887; doi:10.3389/fpls.2015.00576)
Supplement: Supplementary file 3 [file Image1.PDF]

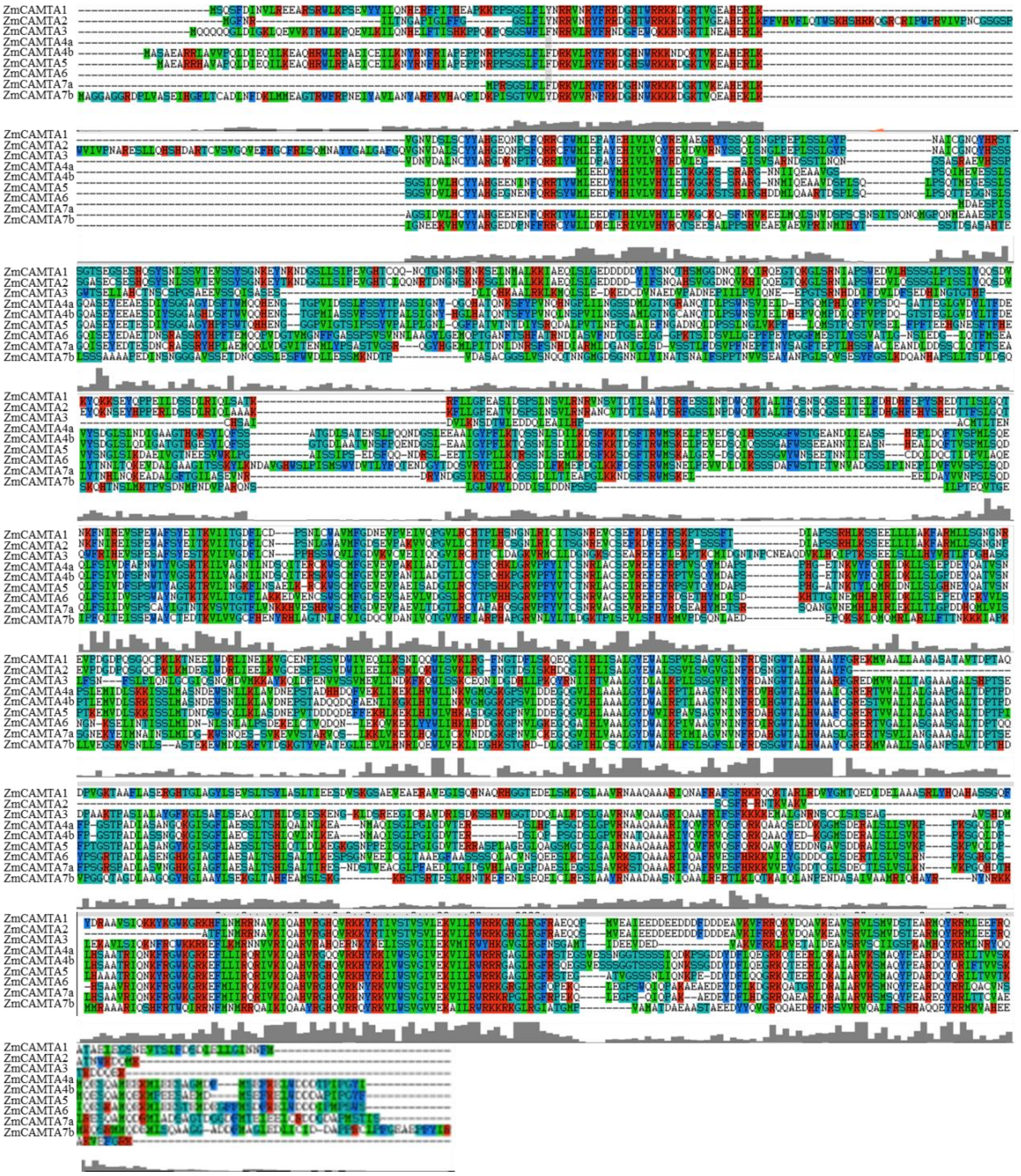

Figure S1 Protein domain analysis of ZmCAMTA family. Alignment of maize ZmCAMTA proteins obtained with the ClustalW program and manual correction. Colorized shading indicates identical and conversed amino acid residues, respectively.
