# Supplementary material for: Identification and expression profiling analysis of calmodulin-binding transcription activator genes in maize (Zea mays L.) under abiotic and biotic stresses
Source: Front Plant Sci. 2015 Jul 28;6:576. doi: 10.3389/fpls.2015.00576 (PMC4516887; doi:10.3389/fpls.2015.00576)
Supplement: Supplementary file 4 [file Image2.PDF]

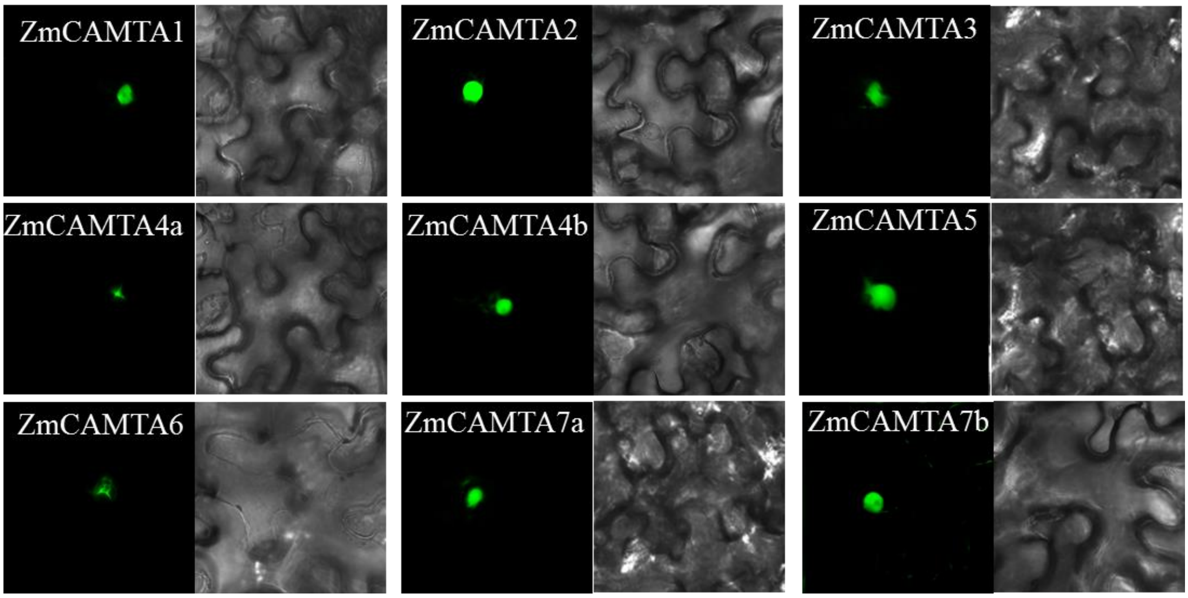

Figure S2 Sub-cellular localization analysis of nine ZmCAMTA proteins. MADS-box protein-GFP fusion proteins transiently were expressed in tobacco epidermis cells. Left to right: green fluorescence of ZmCAMTA-GFP and bright-field.
