# Supplementary material for: Identification and expression profiling analysis of calmodulin-binding transcription activator genes in maize (Zea mays L.) under abiotic and biotic stresses
Source: Front Plant Sci. 2015 Jul 28;6:576. doi: 10.3389/fpls.2015.00576 (PMC4516887; doi:10.3389/fpls.2015.00576)
Supplement: Supplementary file 5 [file Image3.PDF]

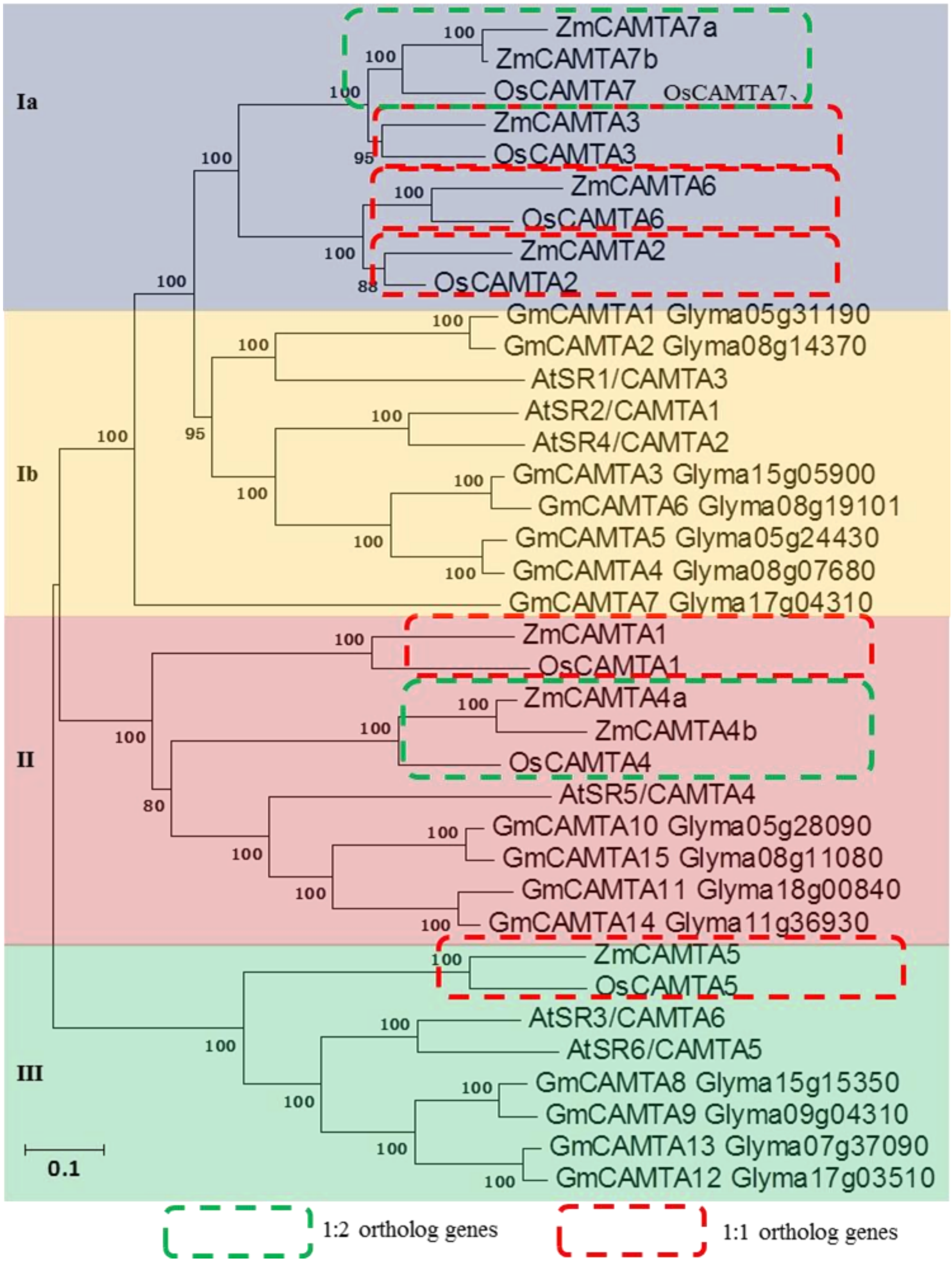

Figure S3 Phylogenetic relationship analysis of *ZmCAMTA* gene. Nine maize CAMTAs were used to build this phylogenetic tree with Maximum likelihood method. Amino acid sequences of these different plants CAMTA proteins were used for analysis. Bootstrap values are presented for all branches.
