# Supplementary material for: Identification and expression profiling analysis of calmodulin-binding transcription activator genes in maize (Zea mays L.) under abiotic and biotic stresses
Source: Front Plant Sci. 2015 Jul 28;6:576. doi: 10.3389/fpls.2015.00576 (PMC4516887; doi:10.3389/fpls.2015.00576)
Supplement: Supplementary file 6 [file Image4.PDF]

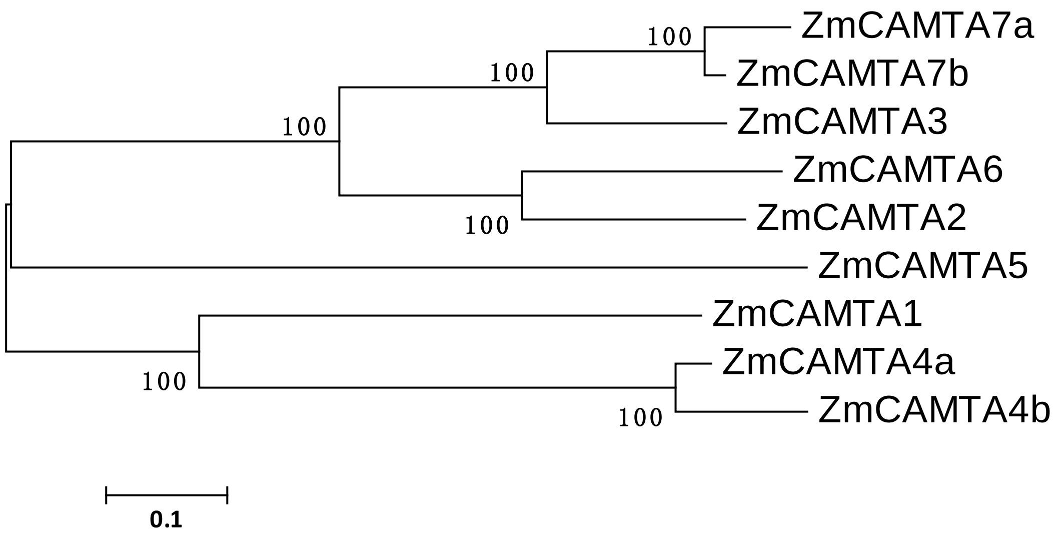

Figure S4 Phylogenetic relationship analysis of *CAMTA* gene family in maize. Nine maize CAMTAs were used to build this phylogenetic tree. Amino acid sequences of these CAMTA proteins were used for analysis. Bootstrap values are presented for all branches.
