# Supplementary material for: Identification and expression profiling analysis of calmodulin-binding transcription activator genes in maize (Zea mays L.) under abiotic and biotic stresses
Source: Front Plant Sci. 2015 Jul 28;6:576. doi: 10.3389/fpls.2015.00576 (PMC4516887; doi:10.3389/fpls.2015.00576)
Supplement: Supplementary file 7 [file Image5.PDF]

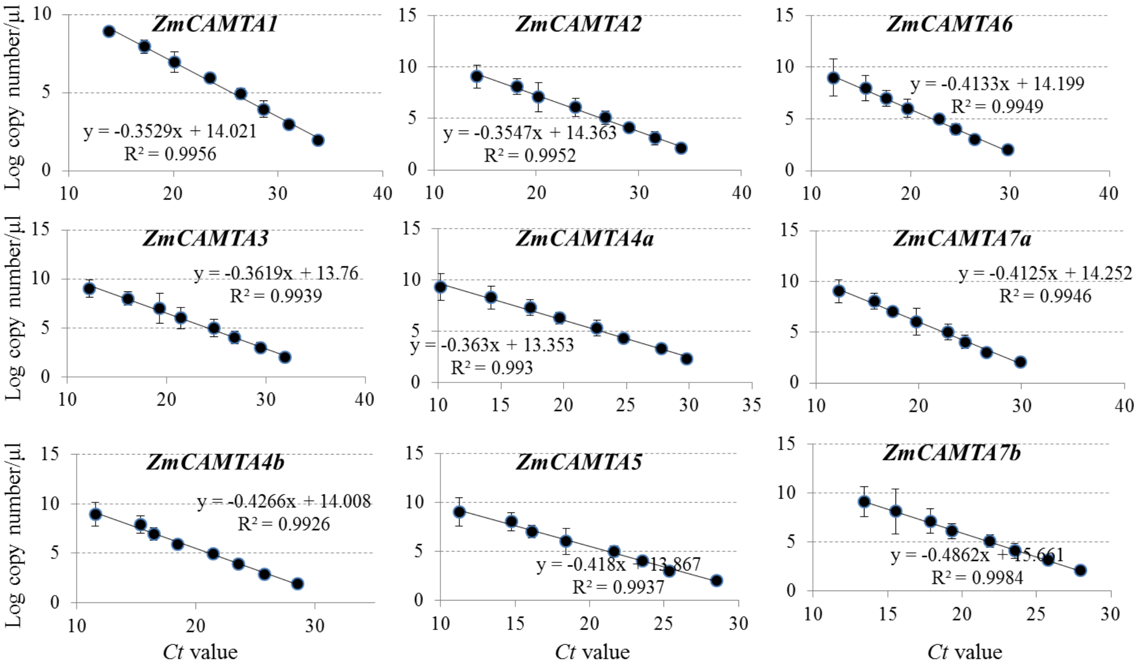

Figure S5 Standard curve of nine *ZmCAMTA* genes. Copy number for each *ZmCAMTA* PCR product was determined spectrophotometrically and diluted serially from 10^1 and 10^8 copies/µL cDNA for use as standard control. Standard curve for each target gene quantification was analyzed by Excel Software 2010.
