# Supplementary material for: Identification and expression profiling analysis of calmodulin-binding transcription activator genes in maize (Zea mays L.) under abiotic and biotic stresses
Source: Front Plant Sci. 2015 Jul 28;6:576. doi: 10.3389/fpls.2015.00576 (PMC4516887; doi:10.3389/fpls.2015.00576)
Supplement: Supplementary file 8 [file Image6.PDF]

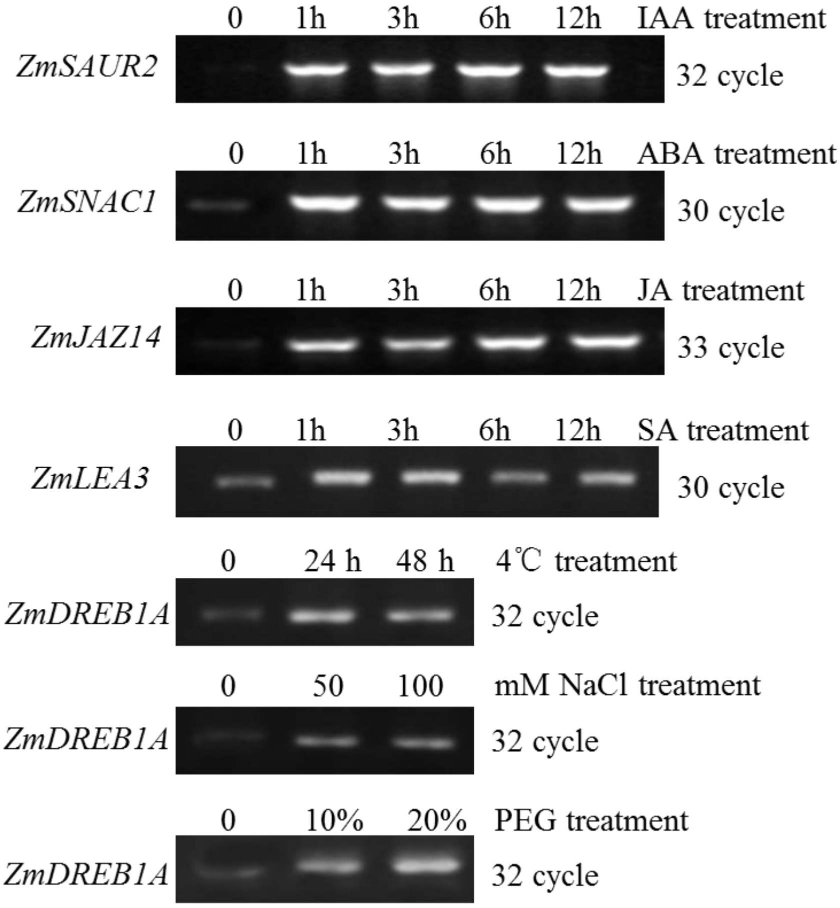

Figure S6 Expression of stress inducible marker genes under different treatments. For hormone treatments, *ZmSAUR2* (GRMZM2G156470) was used as a marker gene for IAA treatment; *ZmSNAC1* (GRMZM2G347043) was used as a marker gene for ABA treatment; *ZmJAZ14* (GRMZM2G064775) was used as a marker gene for JA treatment; *ZmLEA3* (GRMZM2G096475) was used as a marker gene for SA treatment. A well characterized abiotic stress inducible marker gene, *ZmDREB1A* (GRMZM2G124037), was used as control for validating abiotic stresses conditions, including salt, drought and cold. All the primer sequences were listed in Table S1.
