# Supplementary material for: Identification and expression profiling analysis of calmodulin-binding transcription activator genes in maize (Zea mays L.) under abiotic and biotic stresses
Source: Front Plant Sci. 2015 Jul 28;6:576. doi: 10.3389/fpls.2015.00576 (PMC4516887; doi:10.3389/fpls.2015.00576)
Supplement: Supplementary file 9 [file Image7.PDF]

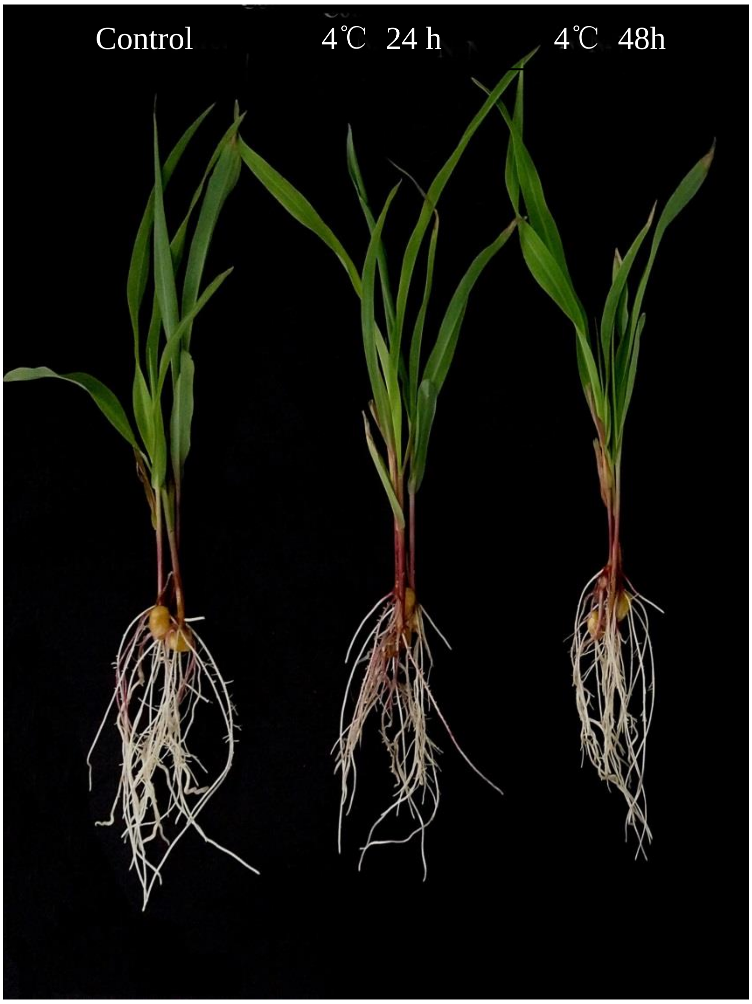

Figure S7 The phenotypic alterations of maize seedlings under cold treatments. For cold treatment, the seedlings were put into a 4 ℃ growth chamber for 24 h and 48 h, and the untreated seedlings were used as control treatment.
