# Supplementary material for: Identification and expression profiling analysis of calmodulin-binding transcription activator genes in maize (Zea mays L.) under abiotic and biotic stresses
Source: Front Plant Sci. 2015 Jul 28;6:576. doi: 10.3389/fpls.2015.00576 (PMC4516887; doi:10.3389/fpls.2015.00576)
Supplement: Supplementary file 10 [file Image8.PDF]

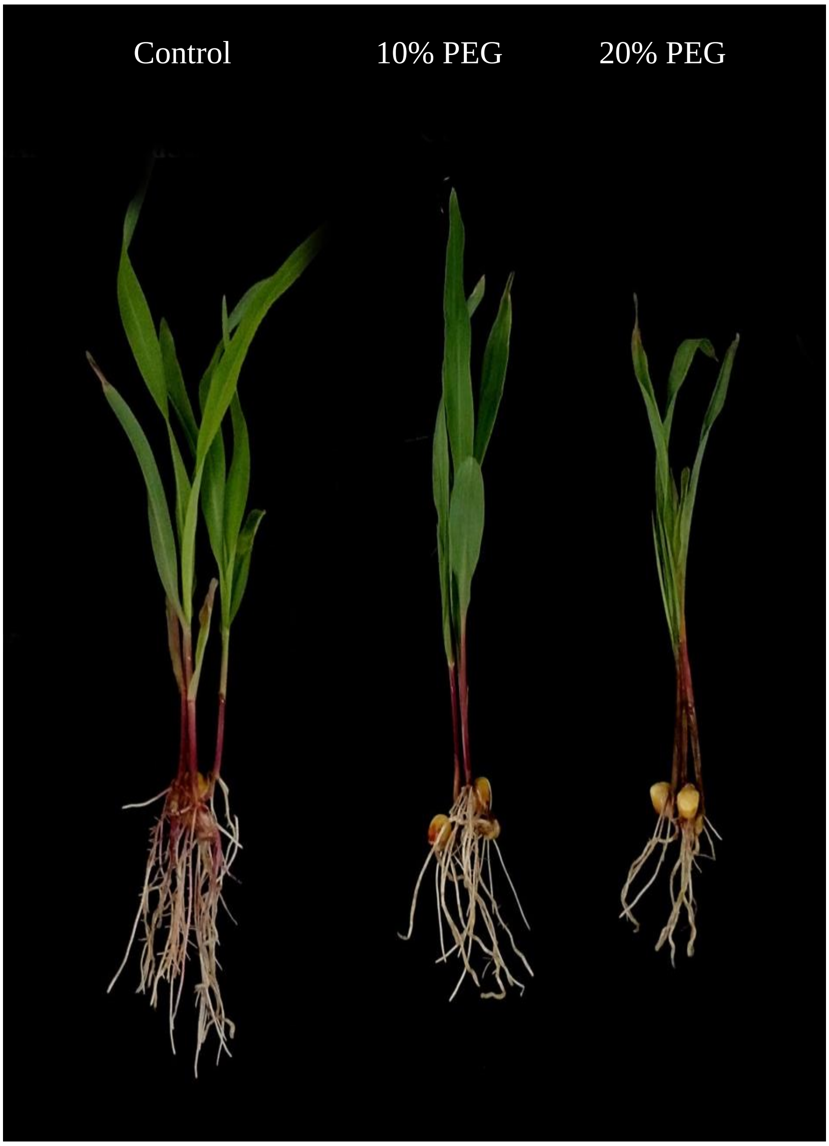

Figure S8 The phenotypic alterations of maize seedlings under PEG treatments. For drought stress treatment, the roots of maize seedlings were soaked in nutrient solution with 10% and 20% polyethylene glycol (PEG) 6000 for four days, and the untreated seedlings were used as control treatment.
