# Supplementary material for: Identification and expression profiling analysis of calmodulin-binding transcription activator genes in maize (Zea mays L.) under abiotic and biotic stresses
Source: Front Plant Sci. 2015 Jul 28;6:576. doi: 10.3389/fpls.2015.00576 (PMC4516887; doi:10.3389/fpls.2015.00576)
Supplement: Supplementary file 11 [file Image9.PDF]

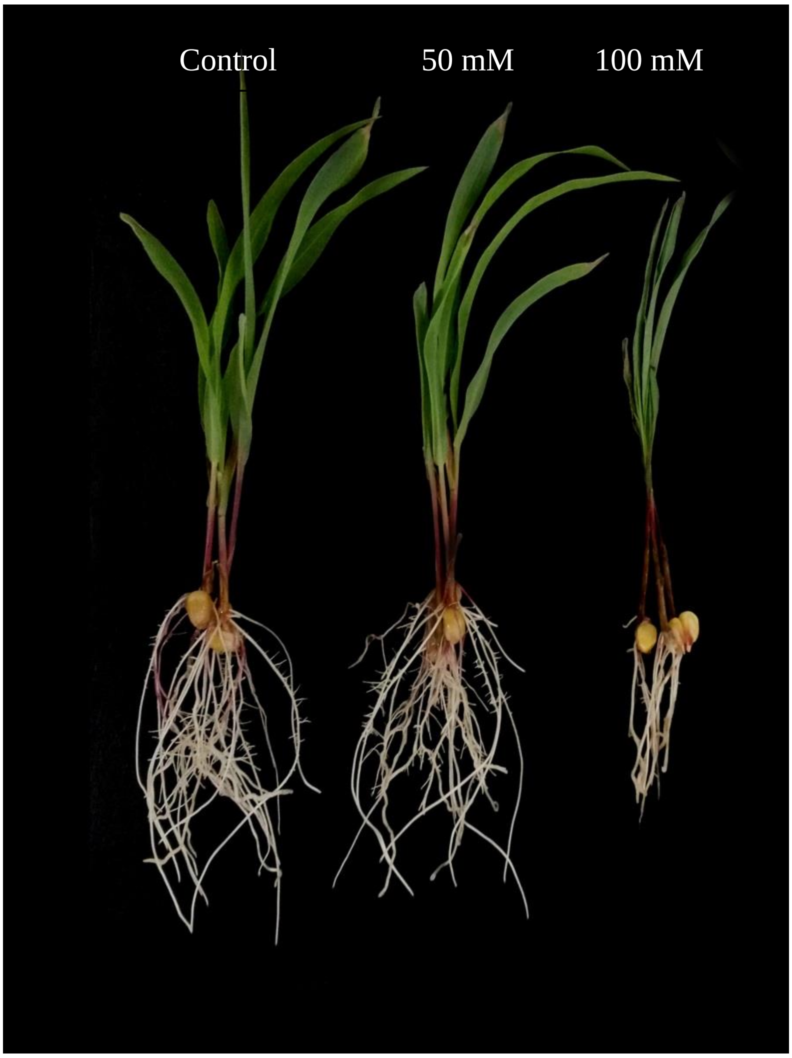

Figure S9 The phenotypic alterations of maize seedlings under NaCl treatments. For salt stress treatment, the roots of maize seedlings were soaked in nutrient solution containing 50 mM and 100 mM NaCl for four days and the untreated seedlings were used as control treatment.
