# Supplementary material for: Identification and expression profiling analysis of calmodulin-binding transcription activator genes in maize (Zea mays L.) under abiotic and biotic stresses
Source: Front Plant Sci. 2015 Jul 28;6:576. doi: 10.3389/fpls.2015.00576 (PMC4516887; doi:10.3389/fpls.2015.00576)
Supplement: Supplementary file 12 [file Image10.PDF]

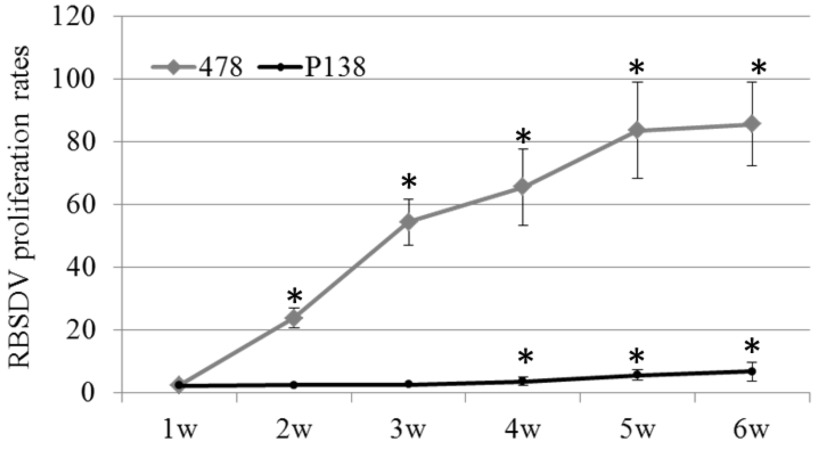

Figure S10 Changes in RBSDV proliferation rates. QRT-PCR was used to calculate the RBSDV proliferation rates in the susceptible maize inbred ‘478’ and the resistant inbred ‘P138’. The significant differences were indicated by an asterisk.
